# Phytofabrication and characterization of *Alchornea cordifolia* silver nanoparticles and evaluation of antiplasmodial, hemocompatibility and larvicidal potential

**DOI:** 10.3389/fbioe.2023.1109841

**Published:** 2023-02-28

**Authors:** Loick Pradel Kojom Foko, Joseph Hawadak, Vaishali Verma, Philippe Belle Ebanda Kedi, Carole Else Eboumbou Moukoko, Raghavendra Kamaraju, Veena Pande, Vineeta Singh

**Affiliations:** ^1^ Parasite and Host Biology Group, ICMR-National Institute of Malaria Research, Dwarka, New Delhi, India; ^2^ Department of Biotechnology, Kumaun University, Nainital, Uttarakhand, India; ^3^ Vector Biology Group, ICMR-National Institute of Malaria Research, Dwarka, New Delhi, India; ^4^ Department of Animal Organisms, Faculty of Sciences, The University of Douala, Douala, Cameroon; ^5^ Nanosciences African Network, iThemba LABS-National Research Foundation, Cape Town, South Africa; ^6^ Laboratory of Innovative Nanostructured Material (NANO: C), Faculty of Medicine and Pharmaceutical Sciences, The University of Douala, Douala, Cameroon; ^7^ Department of Biological Sciences, Faculty of Medicine and Pharmaceutical Sciences, The University of Douala, Douala, Cameroon; ^8^ Malaria Research Unit, Centre Pasteur Cameroon, Yaoundé, Cameroon; ^9^ Laboratory of Parasitology, Mycology and Virology, Postgraduate Training Unit for Health Sciences, Postgraduate School for Pure and Applied Sciences, The University of Douala, Douala, Cameroon

**Keywords:** *Alchornea cordifolia*, silver nanoparticles, green synthesis, characterization, hemocompatibility, biocidal activities, Plasmodium falciparum, culicidae mosquitoes

## Abstract

**Purpose:** The recent emergence of *Plasmodium falciparum* (*Pf*) parasites resistant to current artemisinin-based combination therapies in Africa justifies the need to develop new strategies for successful malaria control. We synthesized, characterized and evaluated medical applications of optimized silver nanoparticles using *Alchornea cordifolia* (AC-AgNPs), a plant largely used in African and Asian traditional medicine.

**Methods:** Fresh leaves of *A. cordifolia* were used to prepare aqueous crude extract, which was mixed with silver nitrate for AC-AgNPs synthesis and optimization. The optimized AC-AgNPs were characterized using several techniques including ultraviolet-visible spectrophotometry (UV-Vis), scanning/transmission electron microscopy (SEM/TEM), powder X-ray diffraction (PXRD), selected area electron diffraction (SAED), energy dispersive X-ray spectroscopy (EDX), Fourier transformed infrared spectroscopy (FTIR), dynamic light scattering (DLS) and Zeta potential. Thereafter, AC-AgNPs were evaluated for their hemocompatibility and antiplasmodial activity against *Pf* malaria strains 3D7 and RKL9. Finally, lethal activity of AC-AgNPs was assessed against mosquito larvae of *Anopheles stephensi*, *Culex quinquefasciatus* and *Aedes aegypti* which are vectors of neglected diseases such as dengue, filariasis and chikungunya.

**Results:** The AC-AgNPs were mostly spheroidal, polycrystalline (84.13%), stable and polydispersed with size of 11.77 ± 5.57 nm. FTIR revealed the presence of several peaks corresponding to functional chemical groups characteristics of alkanoids, terpenoids, flavonoids, phenols, steroids, anthraquonones and saponins. The AC-AgNPs had a high antiplasmodial activity, with IC_50_ of 8.05 μg/mL and 10.31 μg/mL against 3D7 and RKL9 *Plasmodium falciparum* strains. Likewise, high larvicidal activity of AC-AgNPs was found after 24 h- and 48 h-exposure: LC_50_ = 18.41 μg/mL and 8.97 μg/mL (*Culex quinquefasciatus*), LC_50_ = 16.71 μg/mL and 7.52 μg/mL (*Aedes aegypti*) and LC_50_ = 10.67 μg/mL and 5.85 μg/mL (*Anopheles stephensi*). The AC-AgNPs were highly hemocompatible (HC_50_ > 500 μg/mL).

**Conclusion:** In worrying context of resistance of parasite and mosquitoes, green nanotechnologies using plants could be a cutting-edge alternative for drug/insecticide discovery and development.

## 1 Introduction

Nanotechnology has profoundly changed several aspects of human life through technological and health advances made in sectors such as new technologies, energy, cosmetics and health. This term encompasses a set of activities from development research to evaluation of materials sized 1–100 nm—also known as nanomaterials (Subedi, 2013; Bayda et al., 2020). In developed countries such as the United States of America, Japan and China, nanotechnologies are greatly funded, studied and evaluated for their ability to solve diverse problems (Dong et al., 2016; Qiu, 2016). In developing countries, studies are more focused on green nanotechnologies which rely on the development of nanomaterials using living organisms such as plants and microorganisms (Kojom Foko et al., 2019; Kojom Foko et al., 2021).

Green nanotechnologies are very attractive as these are cheaper to implement, safer and eco-friendly as compared to their chemical and physical counterparts (Tran et al., 2013; Gahlawat and Choudhury, 2019). Indeed, chemical and physical methods are time-consuming, costly and request reagents which are harmful to humans and environment (Shakeel et al., 2016). By blending living organisms (e.g., fungi, viruses, bacteria, alga, plants) or derived products with metal source, green metallic nanoparticles (MNPs) are synthesized and then can be tested for different biological and non-biological activities (Honary et al., 2013; Kojom Foko et al., 2019; Bayda et al., 2020; Chugh et al., 2021; Araújo et al., 2022). Also, the utilization of plants is more advantageous than with microorganisms due to increased risk of biohazard and cost to isolate, purify and maintain microbial cultures (Kalishwaralal et al., 2010; Shakeel et al., 2016).

Regarding biological activities, many studies reviewed biocidal potential of green MNPs against non-communicable diseases (e.g., diabetes, cancer), oxidative stress, diverse pathogens (e.g., bacteria, viruses), and disease vectors (e.g., mosquitoes, ticks) (Benelli et al., 2017; Patil and Chandrasekaran, 2020; Araújo et al., 2022). Roughly, plant-based MNPs show a high biocidal potential, and thus were proposed as new avenues for control of infectious diseases, especially mosquito-borne diseases (e.g., malaria, dengue and chikungunya) for which current control methods are jeopardized due to i) their toxicity to humans and environment, and ii) emergence and spread of drug-resistant parasites and insecticide-resistant mosquitoes (Kojom Foko et al., 2019; Kojom Foko et al., 2021). Thus, synthesis of plant-based MNPs could be interesting to develop new drugs and insecticides to control and eliminate mosquito-borne diseases. Malaria is the predominant vector-borne disease globally with an estimated 247 million cases and 619,000 deaths in 2021 (World Health Organization, 2022). Africa bears the bulk of this global malaria burden, with children under 5 years of age and pregnant women being most vulnerable groups (Dongang Nana et al., 2022; World Health Organization, 2022). Resistance of pathogens and mosquito vectors is a great threat to malaria control and elimination efforts (Arya et al., 2021). Recent studies pointed out independent emergence of malaria parasites resistant to current most effective antimalarial drugs (i.e., artemisinin-based combination therapies - ACTs) in two African countries (Rwanda and Uganda) (Uwimana et al., 2021, 2020; Balikagala et al., 2021).

There is paucity of data on biological activities of green MNPs in Cameroon where vector-borne diseases such as malaria are causes of concern (Lehman et al., 2018; Antonio-Nkondjio et al., 2019; Mbohou et al., 2019). In the present study, silver NPs were synthesized using leaves of *Alchornea cordifolia* (AC-AgNPs), optimized, characterized and evaluated for hemocompatibility and lethal activity against *Plasmodium falciparum*—*Pf* (the main and deadliest human malaria species) (Kojom Foko et al., 2021; Kojom Foko et al., 2022b; World Health Organization, 2022), and three mosquito species, i.e., *Anopheles stephensi*, *Culex quinquefasciatus* and *Aedes aegypti*, involved in human transmission of parasites and viruses (dengue, Zika, malaria and lymphatic filariasis) (Benelli et al., 2017; Wang et al., 2017; Patil and Chandrasekaran, 2020). *Alchornea cordifolia* Schumach. *and* Thonn.) Müll*.* Arg*.* (Euphorbiaceae) is largely distributed in sub-Saharan African countries (e.g., Cameroon, Ghana, Nigeria) where its leaves and root bark are traditionally used by populations for nutritional purposes and treating several infectious and inflammatory ailments such as rheumatism, pain and arthritis (Ngaha Njila et al., 2016; Cesar et al., 2017).

## 2 Materials and methods

### 2.1 Study design

**FIGURE 1:**
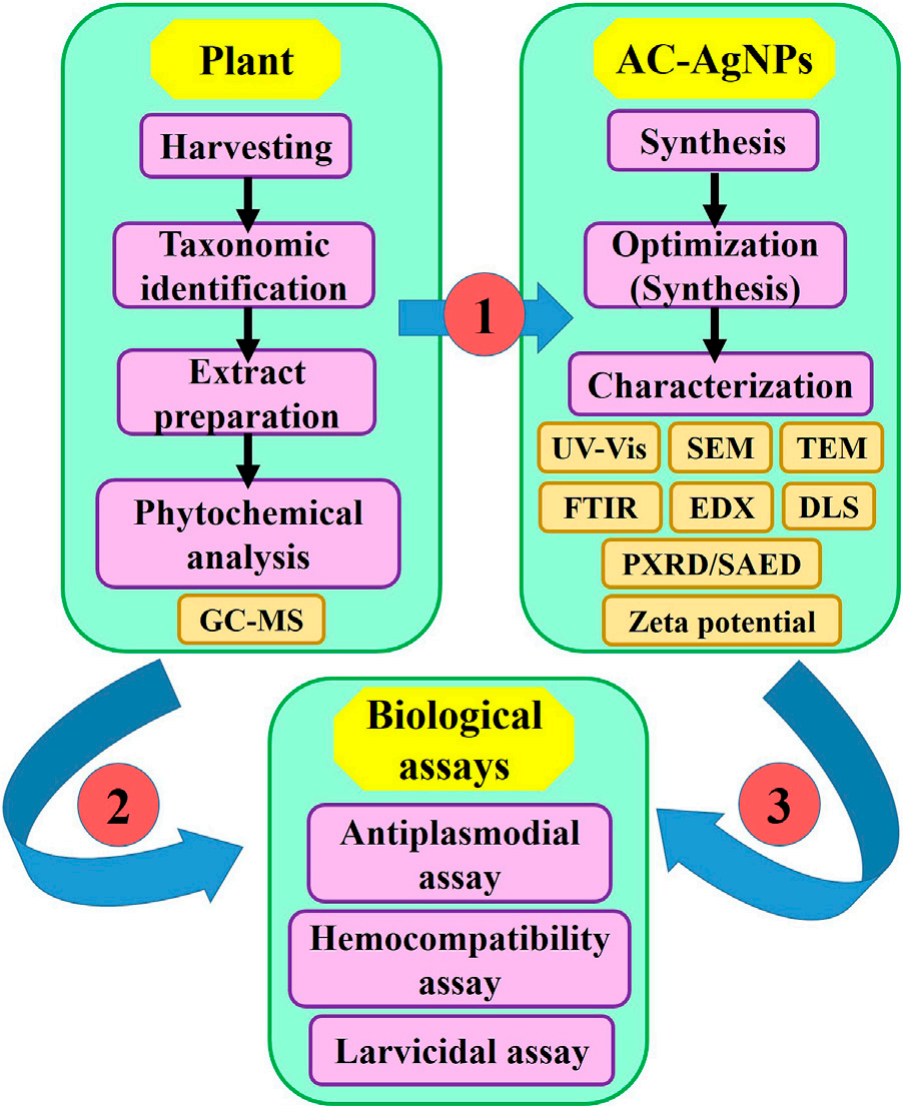
Flowchart depicting the study design. AC-AgNPs, *Alchornea cordifolia* silver nanoparticles; GC-MS, Gas chromatography—Mass spectrometry; DLS, Dynamic light scattering; EDX, Energy dispersive X-ray spectroscopy; FTIR, Fourier transformed infrared spectroscopy; PXRD, Powder X-ray diffraction; SAED, Selected area electron diffraction; SEM, Scanning electron microscopy; TEM, Transmission electron microscopy; UV-Vis, Ultraviolet—Visible spectrophotometry.

This was an experimental study aimed at determining antiplasmodial, hemocompatibility and larvicidal potential of biosynthesized silver NPs using *A. cordifolia* leaves. The plant was harvested and authenticated taxonomically. Crude extract of *A. cordifolia* leaves (AC-CE) was screened for phytochemical composition and used for AgNPs synthesis. The optimization of AC-AgNPs was made, and the optimized AC-AgNPs were characterized and tested for antiplasmodial, hemocompatibility and larvicidal potential (Figure 1). The study was approved by ethical committee of the National Institute of Malaria Research (NIMR), India (N°PHB/NIMR/EC/2020/55).

### 2.2 Collection and authentication of plant material

**FIGURE 2:**
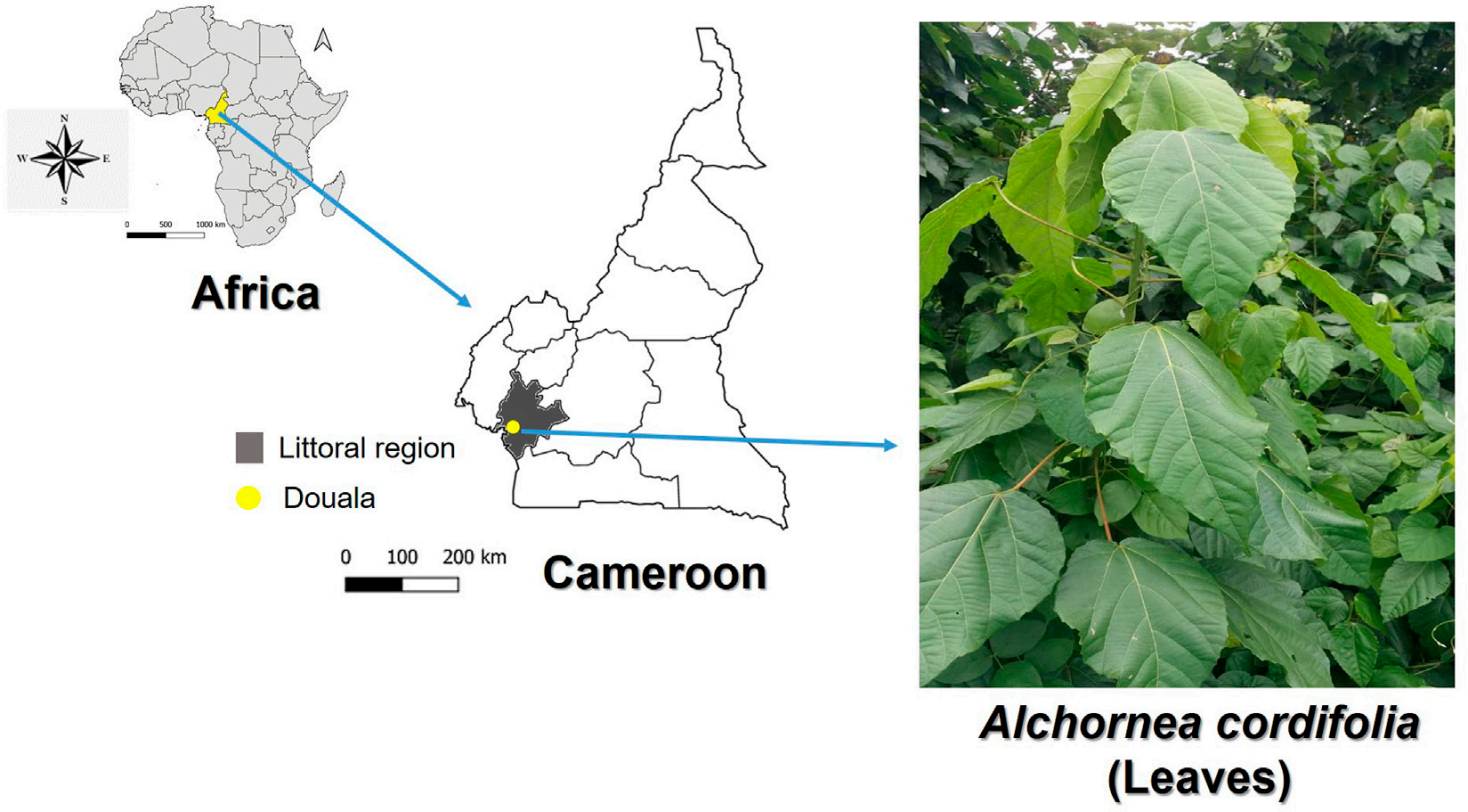
Maps of Africa, Cameroon and Douala (Littoral region) where fresh leaves of *Alchornea cordifolia* (Euphorbiaceae) were harvested. Maps were created using the QGIS software v3.10 (https://qgis.org/en/site/). Photograph of *A. cordifolia* is provided by author PBEK.

Healthy and fresh leaves of *A. cordifolia* (AC) were collected at Faculty of Sciences (FS), main campus, University of Douala (UD), Littoral Region, Cameroon (Figure 2). Malaria is highly prevalent in Cameroon, and *P. falciparum* is the main malaria species. Other species including *Plasmodium vivax*, *Plasmodium ovale spp* have also been reported across the country (Kojom Foko et al., 2021; Kojom Foko et al., 2022a). The taxonomic authentication was done by Dr Tchiengue Barthelemy at Cameroon National Herbarium, Yaounde, in comparison with voucher specimen number 9657/SRF/Cam previously deposited.

### 2.3 Preparation of *A. cordifolia* aqueous extract

About 500 g of fresh *A. cordifolia* leaves were washed with running tap water and distilled water to remove dust and surface contaminant, and thereafter air-dried for 2 weeks at room temperature. The dried material was introduced in an electric grinder to obtain a fine powder. Ten grams of powder was taken in a conical flask containing 100 mL of distilled water, heated at 80°C for 10 min in a water bath under static conditions (Eya’ane Meva et al., 2016). The mixture was allowed to cool at room temperature, and then filtered using a Whatman paper n°1 to remove particulate matter. The filtrate obtained (crude extract, AC-CE) was used to perform phytochemical screening and AC-AgNPs biosynthesis. The AC-CE was not used more than a week following its preparation in order to avoid gradual loss of viability due to long storages (Eya’ane Meva et al., 2016). The AC-CE was lyophilized and stored for biological assays. The yield of extraction of AC-CE was 41% (w/v).

### 2.4 Phytochemical screening of *A. cordifolia* aqueous extract

The AC-CE was subjected to gas chromatography-mass spectrometry (GC-MS) analysis to identify the composition and percentage abundance of phytochemical constituents. The GC-MS was carried out on a Perkin Elmer Turbo Mass Spectrophotometer (Norwalk, CTO6859, NY, United States) which includes a Perkin Elmer Auto sampler XLGC. The column used was a Perkin Elmer Elite-5 capillary column measuring 30 m × 0.25 mm with a film thickness of 0.25 mm composed of 95% dimethyl polysiloxane. The carrier gas used was helium at a flow rate of 1.21 mL/min 1 μL sample injection volume was utilized. The inlet temperature was maintained at 260°C. Oven temperature was programmed initially at 100°C for 2 min, and then programmed to increase to 290°C at a flow rate of 10°C/min (Supplementary Figure S1). The total run time was 39.98 min. The Mass Spectrometry transfer line was maintained at a temperature of 200°C. The source temperature was maintained at 220°C. The GC-MS was analyzed using electron impact ionization at 70 eV. Full scan mode was used to detect analytes. Data were evaluated using total ion count for compound identification and quantification. Measurement of peak areas and data processing were carried out by Turbo-Mass-OCPTVS-Demo SPL software, and spectrums of the components were compared with database of spectrum of known components stored in the GC-MS library.

### 2.5 Phytofabrication and optimization studies of AC-AgNPs

AC-AgNPs were synthesized by blending AgNO_3_ aqueous solution with freshly prepared AC-AE and incubated in dark until color change. The determination of optimal conditions for AC-AgNPs biosynthesis was performed by recording UV-Visible spectra of reaction mixtures after varying four parameters, namely, incubation temperature (35°C–85°C), incubation time (10 min–5 h), AgNO_3_ concentration (0.5–5 mM), and AgNO_3_/AC-CE volume ratio (10–100 µL) as described earlier (Hawadak et al., 2022). Thereafter, the optimized reaction mixture was centrifuged at 15,000 rpm for 10 min, the pellet was washed twice with distilled water and once with 95% ethanol, filtered using sterile syringe filter (MICRO-POR^®^, 0.22 µm), and then lyophilized for further AC-AgNPs characterization and biological assays.

### 2.6 Characterization of AC-AgNPs

The characteristics of green synthesized AC-AgNPs (i.e., surface plasmon resonance-SPR, size, shape, aggregation, functional chemical groups and crystallinity) were determined using several techniques (Figure 1). The formation of AC-AgNPs was monitored by visual inspection of the solution and then followed by UV–Vis spectrum measurement using a double beam spectrophotometer (Model n.o., BRI-2700, BR BIOCHEM Life Sciences Pvt., Ltd., India) operating at 1 nm resolution. Milli Q ultrapure water was used as blank. The selected area electron diffraction (SAED) and powder X-ray diffraction (XRD) were used to determine the physical nature of the AC-AgNPs. The PXRD was made at 45 kv voltage, 40 mA current, 2θ range of 10–80 and speed of 2°/minute (PANanalytical, Xpert Pro model). The PXRD patterns of optimized AC-AgNPs were compared to Joint Committee on Powder Diffraction Standards files (JCPDS 65-2871 and 31-1238). The size, shape and aggregation patterns of AC-AgNPs were determined using scanning electron microscopy—SEM coupled with EDX (Bruker AXS Microanalysis GmbH Berlin, Germany) and transmission electron microscopy—TEM coupled with SAED (TECNAI TF20, Fei, Electron Optics, Oregon, United States) operating at a potential of 20 kv and 200 kv, respectively. The size of NPs was calculated using the Scherrer equation: $D=\frac{K\lambda }{\beta \;\mathrm{Cos}\theta }$ , where D is diameter (nm) of the crystallite (i.e., NPs in this regard), K is the Scherrer constant (range values = 0.68–2.08) depending on shape of nanoparticles (e.g., K = 0.94 for spherical NPs); λ is the X-ray wavelength (in our study PXRD analysis was performed at wavelength for copper, CuK_α_ = 1.5406 Å), β is the line broadening at full width at half maximum (FWHM) which is expressed in radians, and θ is the Bragg’s angle of PXRD-related peaks which is expressed in degrees (Muniz et al., 2016). The atomic composition of the NPs was determined using energy dispersive X-ray (EDX). Fourier transformed infrared spectroscopy (FTIR) was used to determine functional chemical groups capped on the AC-AgNPs surface through potassium bromide method. Sample was grinded with KBr in an infrared path and the spectrum was recorded in the range 400–4000 cm^-1^ using a FTIR spectrophotometer (Perkin Elmer, Frontier Model). Zeta potential and dynamic light scattering (DLS) were performed to evaluate NPs stability and size distribution using particle size analyzer (Zetasizer nano ZS, Malvern Instruments Ltd., U.K.). In practice, zeta potential of ±30 mV is considered as a good indicator of the stability of colloidal suspensions such as NPs while values outside the range indicate phenomena such as flocculation, aggregation and sedimentation (Kojom Foko et al., 2019).

### 2.7 Assessment of antiplasmodial potential of AC-AgNPs

Chloroquine-sensitive 3D7 and chloroquine-resistant RKL9 of *Pf* strains were used for antiplasmodial assays for AC-AgNPs, AC-CE, and chloroquine (CQ). The *Pf* culture was maintained using standard protocols (Trager and Jensen, 1976; Schuster, 2002). Briefly, parasite cultures were maintained in fresh AB positive human erythrocytes suspended at 5% hematocrit in RPMI-1640 culture medium supplemented with L-glutamine and HEPES buffer (0.2% sodium bicarbonate, 0.4% albumax, 50 μg/L hypoxanthine, 200 U/mL penicillin and 200 μg/L streptomycin) and incubated at 37°C under a gas mixture of 1% O_2_, 5% CO_2_ and 94% N_2_. Culture of infected erythrocytes were transferred daily into fresh complete culture medium and checked microscopically for parasite growth.

The *in vitro* evaluation of antiplasmodial activity was performed using culture-adapted *Pf* strains: i) 3D7, sensitive to CQ, artemisinin and its derivatives and ii) RKL9, resistant to CQ. Antimalarial drug screening was done based on SYBR green I-based fluorescence assay as described previously (Smilkstein et al., 2004). Parasite culture (0.5%–0.8%) was synchronized at ring stage with 5% sorbitol. A volume of 100 µL of complete medium were introduced into each well of 96-well microplate, then dilutions were performed for AC-AgNPs and AC-CE (4, 8, 16, 31.25, 62.5, 125, 250 and 500 μg/mL) and CQ (4, 6.25, 12.5, 25, 50, 100, and 200 μg/mL) were added. Ten microliters (10 µL) of synchronized blood were thereafter added in each well, mixed and kept in an incubator at 37°C for 48 h in 96-well flat bottom tissue culture-grade plates under reduced O_2_ atmosphere. Each experiment was replicated thrice. CQ was used as standard drug, while complete medium was considered as negative control. After 48 h-incubation, 100 µL of SYBR Green I in lysis buffer (0.2 µL of the fluorochrome/mL of buffer) was added into each well, mixed gently twice, and the plate was then covered with foil and incubated in a dark chamber for 1 h at room temperature. The buffer lysis consisted of Triton X-100 (0.08% v/v), Tris (20 mM), EDTA (5 mM), and saponin (0.008% wt/v). The fluorescence counts were read using an ELISA reader (Synergy HTX 1708152, Agilent BioTek, Santa Clara, California, United States) with excitation and emission wavelength bands centered at 485 and 530 nm.

### 2.8 Validation of antiplasmodial assay

The SYBR Green based antiplasmodial assay was validated by inspecting microscopic slides of parasite cultures treated with negative control, standard drug, AC-CE and AC-AgNPs (Kaushik et al., 2015; Hawadak et al., 2022). After 48 h-incubation, thick and thin blood films were made, air-dried and stained with 10% Giemsa stain for 20 min. The number of schizonts with ≥2 nuclei out of 200 asexual parasites was noted. Also, fluorescence counts of untreated and treated *Pf* cultures were compared to detect any quenching effect-related measurement artefacts which may due to chemical compounds of AC-AgNPs and AC-CE (Kaushik et al., 2015).

### 2.9 Hemocompatibility investigation

The method described by Wang and others was used to evaluate hemocompatibility of biosynthesized AC-AgNPs (Wang et al., 2010). Human red blood cells (RBCs) were obtained from the ICMR-NIMR malaria parasite bank, washed with incomplete media, and diluted with phosphate-buffered saline (PBS) to obtain a suspension (Hematocrit = 1%). Different concentrations (2, 4, 8, 16, 30, 62.5, 125, 250 and 500 μg/mL) of AC-AgNPs and AC-CE were incubated with RBCs in Eppendorf tubes (20 µL of each concentration in 180 µL blood) at 37°C for 30 min and 24 h at pH of 7.40. The reaction was stopped by placing tubes at 4°C for 15 min. The mixtures were then centrifuged at 3,000 g for 4 min, and 100 µL of supernatant was loaded into a 96-well plate to measure the released hemoglobin at 540 nm (SPECTROstar^*Nano*^, BMG LABTECH GmbH, Ortenberg Germany). Saponin was used as positive control, inducing 100% hemolysis, while PBS was considered as negative control. The experiment was performed in triplicate. RBCs hemolysis at each concentration after 30 min and 24 h was calculated as follows:

$$\%\;hemolysis=\frac{\left(As-ANC\right)}{\left(APC-ANC\right)}\times 100$$

where A_S_, A_NC_ and A_PC_ are the absorbance of the sample, negative control (PBS) and positive control (saponin).

### 2.10 Mosquito rearing

The eggs of *An. stephensi*, *Cx. quinquefasciatus* and *Ae. aegypti* were obtained from NIMR Insectarium, New Delhi, India. The characteristics of mosquitoes used are as follows: *An. stephensi*—laboratory strain collected from Sonepat, Haryana, India (established in 1996; black and brown, malathion-deltamethrin-susceptible and DDT–resistant strain), *Cx. quinquefasciatus*—laboratory strain collected from Sonepat, Haryana, India (established in 1999; selected for permethrin resistance and is resistant to DDT, malathion and deltamethrin), and *Ae. aegypti*—laboratory strain collected from Delhi, India (established in 2006; DDT–malathion-deltamethrin strain). Adult *Ae. aegypti* were derived from batches of 100 eggs in 18 cm × 13 cm × 4 cm trays containing 500 mL of boiled and cooled water in a laboratory maintained at 25°C–29°C temperature and 65%–70% Relative humidity; 12:12 h Light/Dark photoperiod. Eggs were fed daily with TetraBits fish food (Tetra GmbH, Herrenteich, Germany), and late 3rd and 4th instar larvae were used for larval bioassays.

### 2.11 Larvicidal bioassays

The protocol described by the World health Organization (WHO) was used for this experiment (WHO, 2016). Late 3^rd^ and 4^th^ instar larvae were exposed to the AC-AgNPs with different concentrations (0–50 μg/mL). Each concentration was tested in triplicate comprising of 25 larvae placed into plastic bowls (8 cm diameter, 300 mL capacity) containing distilled water. The larval mortality was monitored after 24 h, 48 h and 72 h post-treatment periods, and the lethal concentrations to cause 50%/90% mortality in treated larvae (LC_50_/LC_90_) and percentage mortality after post-treatment periods were calculated as described previously in the WHO procedures (WHO, 2016). Distilled water was used as control. All experiments were performed under laboratory conditions as described above.

### 2.12 Statistical analysis

Data was keyed into an Excel spreadsheet (Microsoft Office, United States) and then exported to statistical package for social sciences v16 (SPSS, IBM, Inc., Chicago, United States), and GraphPad v5.03 (GraphPad PRISM, Inc., San Diego, California, United States) for statistical analysis. Using GraphPad software v8.03 (GraphPad PRISM, Inc., San Diego, CA, United States), fluorescence counts of antiplasmodial assay were used to plot graph of percent inhibition of *Pf* parasite growth against concentrations of AC-AgNPs, AC-CE, and CQ to determine 50% inhibition concentration (IC_50_). The dose/time mortality response data of larvicidal assays was analyzed using log-probit regression model to determine LC_50_ and LC_90_ with their confidence interval at 95% (95% CI). The Abbott’s formula was used to correct mortality rate if comprised between 5% and 20% in the negative control group (Sun and Shepard, 1947). Experiments were considered invalid when mortality rate in negative control group was >20%. Regarding hemocompatibility assay, the amount of NPs required to lyse 50% of RBCs (hemolysis concentration, HC_50_) was determined. Quantitative and qualitative variables were presented as mean ± standard deviation (SD) and percentages, respectively. One-way analysis (ANOVA), McNemar’s and Pearson’s independence chi square tests were used to make comparisons. The level of statistical significance was set at *p*-value <0.05.

## 3 Results

### 3.1 GC-MS analysis

GC-MS chromatogram of AC-CE revealed several peaks which represent different compounds as shown in Supplementary Figure S1. A total of 42 compounds were identified in AC-CE after comparing the peaks with database of spectrum of known components stored in the GC-MS library (Table 1). Two compounds were predominantly represented, namely, 2-hexadecen-1-ol, 3,7,11,15-tetramethyl-, [R-[R*,R*-(E)]]- and phytyl tetradecanoate, with proportions of 25.14% and 14.53%, respectively (Supplementary Figure S2; Table 1).

**TABLE 1:** Phytochemical screening of the AC-AE using GC-MS analysis.

Peak	Retention time	Area (%)	Name of the compounds
1	6.76	2.58	4-Methylmannitol
2	9.22	0.29	Dodecanoic acid, methyl ester
3	9.52	0.48	2(4H)-Benzofuranone, 5,6,7,7a-tetrahydro-4,4,7a-trimethyl-, (R)-
4	10.04	0.57	1-Hexadecene
5	10.99	0.41	8-Pentadecanone
6	11.59	0.36	1,1,4,7-Tetramethyldecahydro-1H-cyclopropa[e]azulene-4,7-diol
7	12.08	1.42	6-Hydroxy-4,4,7a-trimethyl-5,6,7,7a-tetrahydrobenzofuran-2(4H)-one
8	12.31	0.58	1-Nonadecene
9	12.37	0.51	2(4H)-Benzofuranone, 5,6,7,7a-Tetrahydro-6-hydroxy-4,4,7a-trimethyl-
10	12.51	1.00	(S,E)-4-Hydroxy-3,5,5-trimethyl-4-(3-oxobut-1-en-1-yl)cyclohex-2-enone
11	12.77	1.28	Neophytadiene
12	12.84	1.08	2-Pentadecanone, 6,10,14-trimethyl-
13	13.02	0.16	2-hexadecen-1-ol, 3,7,11,15-tetramethyl-, [R-[R*,R*-(E)]]-
14	13.08	0.32	1,2-Benzenedicarboxylic acid, bis(2-methylpropyl) ester
15	13.58	0.60	1,2-Benzenedicarboxylic acid, bis(2-methylpropyl) ester
16	13.69	1.86	Hexadecanoic acid, methyl ester
17	14.22	2.36	n-Hexadecanoic acid
18	14.36	0.22	1-Octadecene
19	14.84	1.99	Hexadecanoic Acid, trimethylsilyl ester
20	15.33	0.43	9,12-Octadecadienoic acid (Z,Z)-, methyl ester
21	15.39	3.64	9,12,15-Octadecatrienoic acid, methyl ester, (Z,Z,Z)-
22	15.51	25.17	2-Hexadecen-1-Ol, 3,7,11,15-Tetramethyl-, [R-[R*,R*-(E)]]-
23	15.62	0.56	Methyl stearate
24	15.99	1.40	Phytol, TMS derivative
25	16.40	0.54	Phytol, acetate
26	17.63	0.21	4,8,12,16-Tetramethylheptadecan-4-olide
27	19.09	1.41	Bis(2-ethylhexyl) phthalate
28	20.31	0.36	Tetracontane
29	21.14	1.70	Squalene
30	21.57	0.40	.alpha.-Tocospiro B
31	21.78	0.52	Hexatriacontane
32	22.52	1.11	9,12-Octadecadienoic Acid (Z,Z)-, 2,2-Dimethyl-1,3-Dioxolan-4-Ylmethyl Ester
33	24.12	0.81	Vitamin E
34	24.84	3.32	Ethanone, 1-(2,3,4,7,8,8a-hexahydro-3,6,8,8-tetramethyl-1H-3a,7-methanoazulen-5-yl)-
35	25.93	3.65	STIGMASTA-5,22-DIEN-3-OL
36	26.87	4.69	.gamma.-Sitosterol
37	27.32	4.03	9,19-Cyclolanostane-3,7.beta.-diol, diacetate (20R,14.beta.)
38	28.25	0.99	9,19-Cyclolanostan-3-ol, 24-methylene-, (3.beta.)-
39	31.55	14.53	Phytyl tetradecanoate
40	35.11	3.40	Methanesulfonic Acid 2-(3-Hydroxy-4,4,10,13,14-Pentamethyl-2,3,4,5,6,7,10,11,12,13,14,15,16,17-Tetradecahydro-1h-Cyclopenta[A]Phenan
41	37.27	3.92	1-Eicosanol
42	37.77	5.12	9,10,12,13-Tetrabromooctadecanoic acid
	Total	100.00	

### 3.2 UV-Vis spectroscopy and AC-AgNPs optimization

**FIGURE 3:**
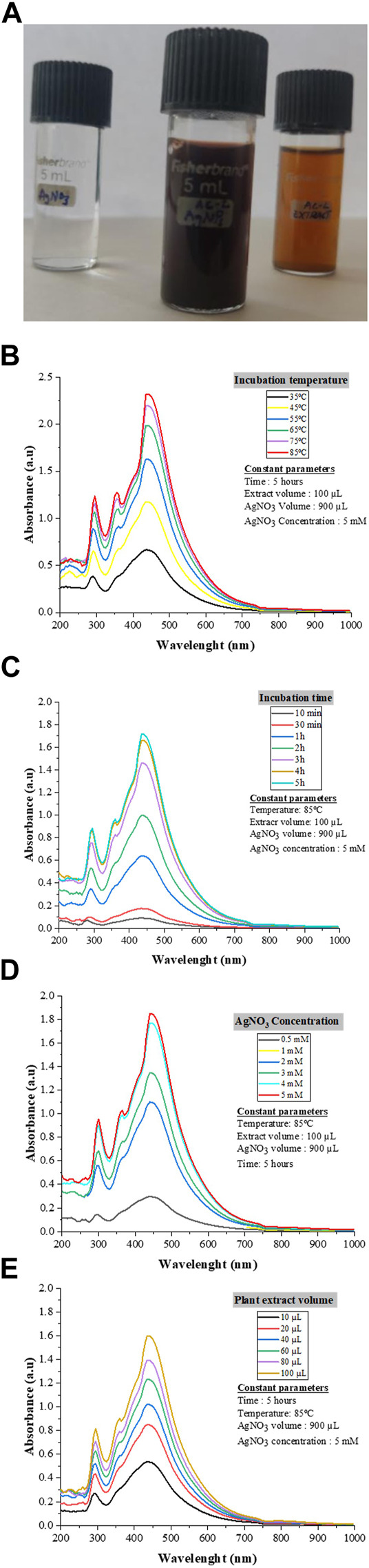
AC-AgNPs solution and UV-Vis findings. Color of AgNO_3_, AC-AgNPs and AC-CE solutions **(A)**. The color change indicates Ag^+^ reduction to elemental nanosilver. UV–visible spectrum of optimized AC-AgNPs for incubation temperature **(B)**, incubation time **(C)**, AgNO_3_ concentration **(D)**, and volume of AC-CE **(E)**. AC-AgNPs, *Alchornea cordifolia* silver nanoparticles; AC-CE, *Alchornea cordifolia* crude extract; AgNO_3_, Silver nitrate; UV-Vis, Ultraviolet—Visible spectrophotometry.

The synthesis of AC-AgNPs was noted after 2 minutes following the incubation of plant extract and AgNO_3_ solution as a dark brown color was observed (Figure 3A). The UV-Vis spectrum analysis revealed a SPR at 445 nm wavelength (Figures 3B–E). The SPR did not change with the variation of four parameters used to optimize AC-AgNPs synthesis (AgNO_3_ concentration, incubation time, incubation temperature and volume of plant extract). In contrast, the amplitude of UV-Vis curves gradually increased with increasing values of each parameter (Figures 3B–E). Thus, the optimization of AC-AgNPs was achieved for the following parameters: 100 µL of fresh plant extract was mixed with 900 µL of AgNO_3_ (5 mM), and then incubated at 85°C for 5 h under static conditions.

### 3.3 Electron microscopy analysis of green AC-AgNPs

**FIGURE 4:**
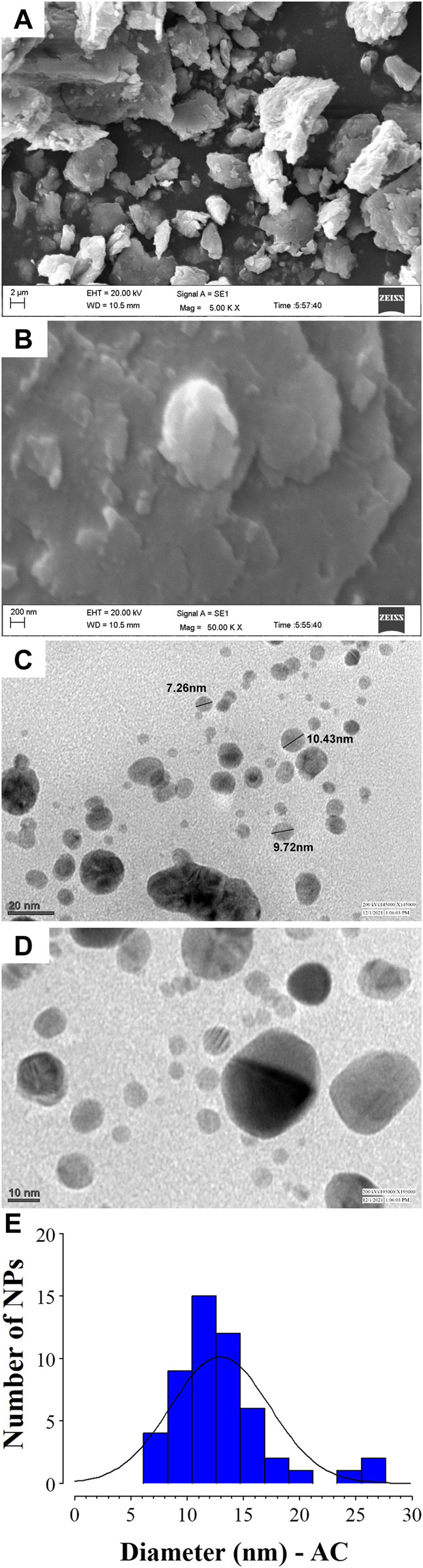
SEM and TEM analysis of AC-AgNPs. SEM images at 5.00 KX **(A)** and 50.00 KX **(B)** of AC-AgNPs. Micrographs of the AC-AgNPs using TEM at 20 nm **(C)** and 10 nm **(D)**, and size distribution of the nanocrystallites **(E)**. AC-AgNPs, *Alchornea cordifolia* silver nanoparticles; SEM, Scanning electron microscopy; TEM, Transmission electron microscopy.

Analysis of SEM and TEM micrographs of AC-AgNPs is depicted in Figure 4. Based on SEM, agglomeration of AC-AgNPs was observed (Figures 4A, B). TEM images of silver colloidal solution exhibited that AC-AgNPs were polydispersed, predominantly spheroidal with various sizes (Figures 4C, D). The size distribution when a section of these NPs is considered is presented in Figure 4E. Following the digitization phase of various images, size distribution using ImageJ software was found to be within 5–25 nm range. The distribution of AC-AgNPs size was large, with mean size ±SD of 10.89 ± 5.67 nm.

### 3.4 PXRD analysis, SAED patterns, and composition of AC-AgNPs

**FIGURE 5:**
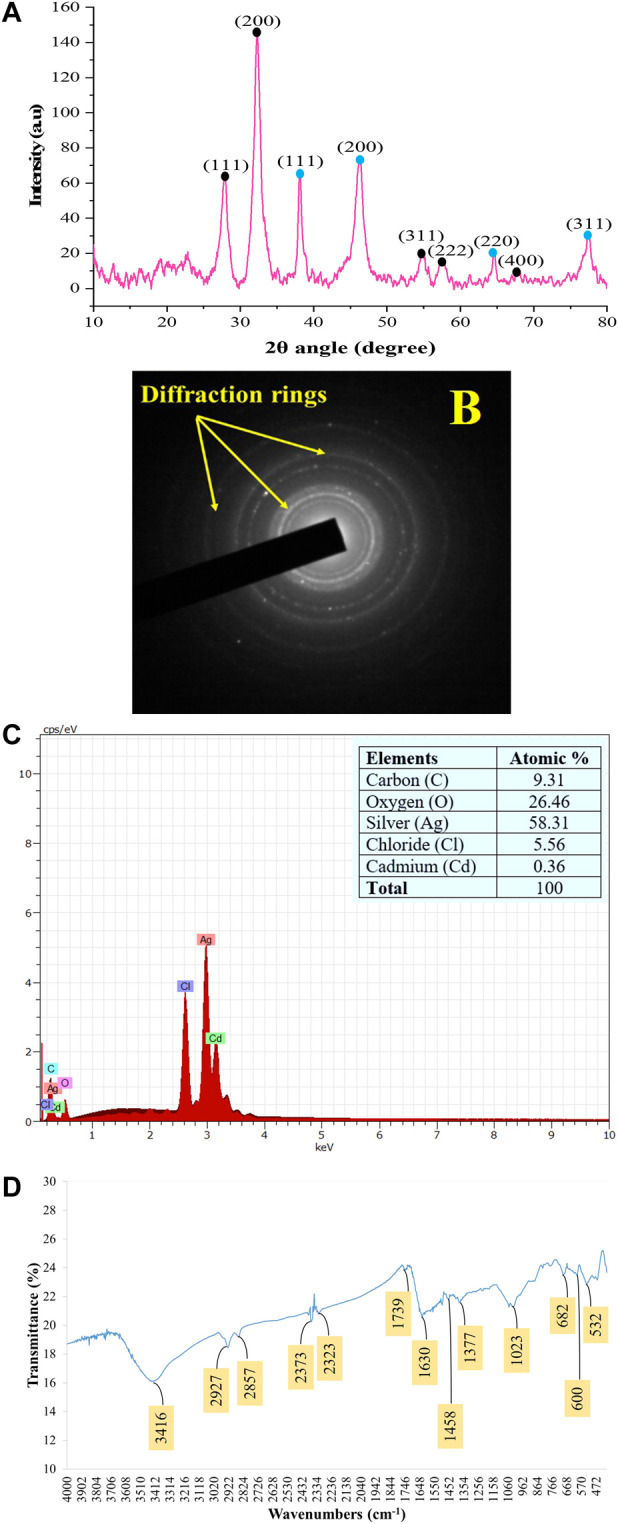
Patterns of the green synthesized AC-AgNPs using PXRD **(A)**, SAED **(B)**, EDX **(C)**, and FTIR **(D)**. In **(A)**, intensity of peaks is presented as arbitrary units (a.u). In **(A)**, peaks with blue and black round shape indicate silver nanocrystals and silver chloride nanocrystals, respectively. AC-AgNPs, *Alchornea cordifolia* silver nanoparticles; EDX, Energy dispersive X-ray spectroscopy; FTIR, Fourier transformed infrared spectroscopy; PXRD, Powder X-ray diffraction; SAED, Selected area electron diffraction.

The PXRD patterns outline that AC-AgNPs are face-centered cubic. The intense and narrow diffraction peaks revealed the formation of pure crystals of silver and silver chloride. The nanosilver crystal peaks obtained at 2θ values of 38.07°, 46.20°, 64.33° and 77.40° which correspond to the (111), (200), (220) and (311) planes of the face-centered cubic (fcc) structures, respectively (JCPDS file 65-2871). Additional peaks corresponding to silver chloride nanocrystallites were observed at 2θ values of 27.8°, 32.2°, 54.8°, 57.4° and 67.4° indexed to (111), (200), (311), (222) and (400) planes, respectively (JCPDS file 31-1238). SAED suggests that the NPs are polycrystalline with diffraction rings associated due to their stacking each other due to their magnetite phase (Figures 5A, B). The crystallinity percentage of AC-AgNPs was 84.13%. Using data from PXRD, the size of silver nanocrystals and silver chloride nanocrystals based on the Scherrer formula was 13.47 ± 5.81 nm and 10.42 ± 3.34 nm, respectively (Table 2). The overall mean size of AC-AgNPs was 11.77 ± 5.57 nm.

**TABLE 2:** Principal characteristic values of the powder X-ray diffractogram of AC-AgNPs.

S.No.	Position (2θ)	Peak amplitude (a.u)	FWHM (2θ)	Cos (θ)	Miller indices (HKL)	Nature	Size (nm)
1	27.83	64.55	1.1219	0.97065	(111)	AgCl	7.62
2	32.31	146.69	1.2155	0.96051	(200)	AgCl	7.11
3	38.15	66.67	0.6446	0.94509	(111)	Ag	13.62
4	46.20	75.30	1.3896	0.91982	(200)	Ag	6.49
5	54.81	19.36	0.9351	0.88778	(311)	AgCl	10.00
6	57.41	16.20	0.7762	0.87710	(222)	AgCl	12.19
7	64.59	20.41	0.4035	0.84531	(220)	Ag	24.33
8	67.41	10.75	0.6573	0.83191	(400)	AgCl	15.17
9	77.40	31.95	1.1259	0.78043	(311)	Ag	9.44

a.u, Arbitrary units; FWHM, full width at half maximum; Cos, Cosinus.

The EDX profile of AC-AgNPs showed a strong signal due to silver atom (Ag) which was involved in AC-AgNPs at a percentage of 58.31%. Other signals due to chlorine (Cl), cadmium (Cd), carbon (C) and oxygen (O) were also observed at 5.56%, 0.36%, 9.31% and 26.46%, respectively (Figure 5C). The identity of functional chemical groups at the interface of AC-AgNPs were determined using FTIR which revealed strong signals at 3,416 cm^−1^, 1630 cm^−1^, and 1023 cm^−1^ which are characteristics of alcohols (O-H stretch), alkenes (C=O stretch) and alkyl and Aryl Halides (C-F stretch), respectively. Smaller signals corresponding to alkanes (C-H stretch) at 2927 cm^−1^, alkanes/aldehydes/alkenes (C-H stretch, C-O stretch) at 2857/1739 cm^−1^, nitriles (C≡N stretch) at 2373/2323 cm^−1^, aromatic compounds (C=C stretch) at 1458 cm^−1^, and nitro compounds (NO_2_ stretch) at 1377 cm^−1^ were also seen (Figure 5D; Table 3).

**TABLE 3:** Functional groups at a given wavenumber for the FTIR spectra of AC-AgNPs.

Absorption (cm^-1^)	Appearance	Functional groups	Compound class
3,416	Medium	N-H stretching	Primary amine
2,927	Sharp	C-H stretching	Alkane
2,857	Medium	C-H stretching	Alkane
2,373	Sharp	O=C=O stretching	Carbon dioxide
		C≡N stretching	Nitriles
2,323	Weak	O=C=O stretching	Carbon dioxide
		C≡N stretching	Nitriles
1,739	Sharp	C=O stretching	Ester, Aldehyde, Saturated aliphatic, or δ-lactone
1,630	Medium	C=C stretching	Conjugated alkene
1,458	Medium	C-H bending	Alkane (methylene or methyl group)
1,377	Medium	C-H bending	Aldehyde or Alkane (gem dimethyl)
1,023	Sharp	C-O stretching	Alcohol, Ether, Carboxylic acids
682	sharp	C=C bending	Alkene or Aromatics
600	sharp	C-I stretching	Halo compound
532	Sharp	C-Br stretching	Alkyl halides

### 3.5 Zeta potential and DLS

The stability of AC-AgNPs was determined using zeta potential, and the analysis revealed a zeta potential value of −18.1 mV which outlines a good stability (Supplementary Figure S3). On analysis of DLS results, the AC-AgNPs had a mean size ±SD of 89.77 ± 16.50 nm, with polydispersity index of 0.242 (Supplementary Figure S4).

### 3.6 Antiplasmodial assays

**FIGURE 6:**
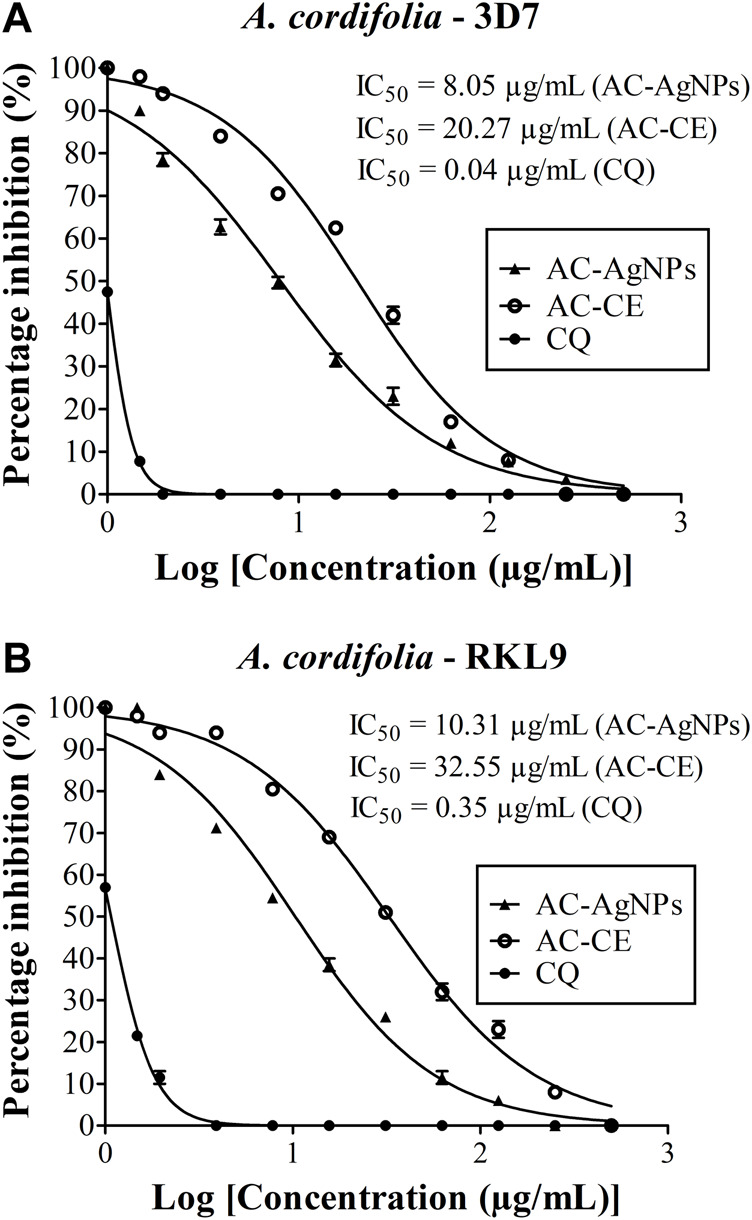
Antiplasmodial activity of AC-CE and AC-AgNPs against laboratory **(A)** *P. falciparum* 3D7 and **(B)** RKL9 strains. AC-AgNPs, *Alchornea cordifolia* silver nanoparticles; AC-CE, *Alchornea cordifolia* crude extract; CQ, Chloroquine; IC_50_, 50% Inhibition concentration. CQ was used as standard drug. Reference *P. falciparum* strains 3D7 (CQ-sensitive) and RKL9 (CQ-resistant) were used. The experiments were triplicated.

High antiplasmodial activity was found for AC-AgNPs against 3D7 (CQ-sensitive) and RKL9 (CQ-resistant) *Pf* strains. Based on IC_50_ values, AC-AgNPs exhibited higher antiplasmodial activity as compared to that of AC-CE irrespective of plasmodial strain, and differences were statistically significant (*p* < 0.0001): 8.05 μg/mL vs*.* 20.27 μg/mL for 3D7, and 10.31 μg/mL vs*.* 32.55 μg/mL for RKL9. The standard drug CQ exhibited IC_50_ values of 0.04 μg/mL and 0.35 μg/mL against *Pf* strains 3D7 and RKL9 (Figures 6A, B). The SYBR green assay findings were supported by microscopic data. AC-AgNPs and AC-CE elicited no quenching effects as no statistically significant difference was found between fluorescence counts of NPs, standard drug and plant extract (Supplementary Figure S5).

### 3.7 Hemolysis induced by the green AC-AgNPs

**FIGURE 7:**
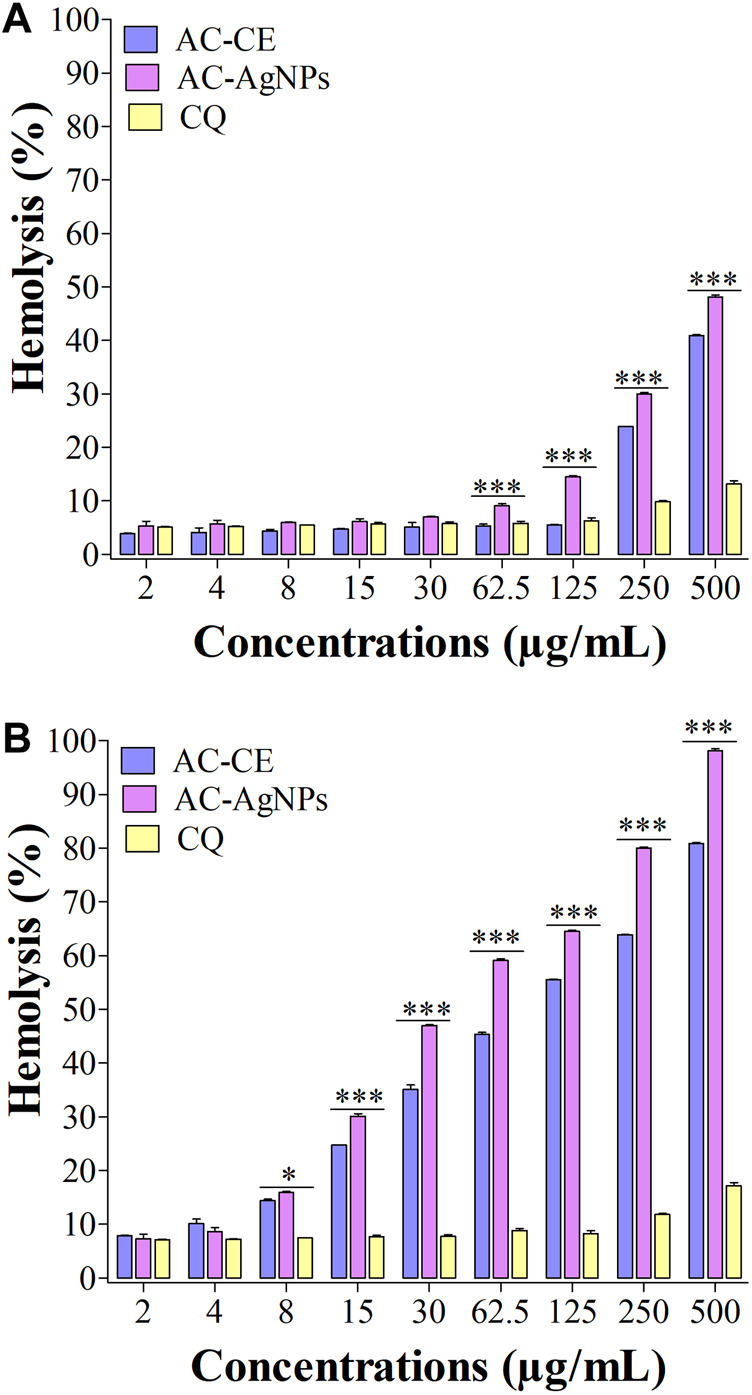
Hemolysis effect of AC-CE and AC-AgNPs after 30 min **(A)** and 24 h **(B)**. AC-AgNPs, *Alchornea cordifolia* silver nanoparticles; AC-CE, *Alchornea cordifolia* crude extract; CQ, Chloroquine; IC_50_, 50% Inhibition concentration. The experiment was performed in triplicate. Saponin was used as positive control and PBS as negative control. Statistically significant at *p < 0.05, **p < 0.01 and ***p < 0.0001.

We have noted that hemolysis rates were dependent on substance, dose and time (Figures 7A, B). After 30 min, hemolysis rates elicited by AC-AgNPs and AC-CE were significantly higher than that of CQ at doses ≥62.5 μg/mL. At these concentrations (125–500 μg/mL), hemolysis rates ranged from 6.25%–13.15% for CQ, 14.55%–48.14% for AC-AgNPs, and 5.50%–40.95% for AC-CE (Figure 7A). To be noted, HC_50_ was not achieved after 30-min incubation as hemolysis rate was below 50% at 500 μg/mL. After 24 hour-incubation, hemolysis rates increased for all substances tested, with the highest values in AC-AgNPs-treated samples (maximum hemolysis of 98.14% at 500 μg/mL). Statistically significant difference between AC-AgNPs, AC-CE, and CQ were seen at doses ≥8 μg/mL (Figure 7B).

### 3.8 Toxic effect of the AC-AgNPs against mosquito species

Mortality of *Cx. quinquefasciatus*, *Ae. aegypti* and *An. stephensi* larval stages was followed 24h, 48h and 72 h after treatment with AC-AgNPs. Mortality rates of the three mosquito species increased as a function of time and concentration (Supplementary Figure S6). After 48 h incubation, larval mortality rates were 100% at doses 23.5 μg/mL for *Cx. quinquefasciatus*, 20 μg/mL for *Ae. aegypti*, and 15 μg/mL for *An. stephensi* (Supplementary Figure S6). AC-AgNPs were more lethal against *An. stephensi* regardless of exposure time, with LC_50_ values of 10.67 μg/mL and 5.85 μg/mL at 24 h- and 48 h-exposure, respectively. These values were 16.71 μg/mL and 7.52 μg/mL for *Ae. aegypti*; 18.41 μg/mL and 8.97 μg/mL for *Cx. quinquefasciatus*, respectively. Regardless of exposure time, the larvicidal activity of AC-AgNPs was much higher than that of AC-CE for which LC_50_ of 231.41 μg/mL, 110.33 μg/mL and 53.15 μg/mL against *Cx. quinquefasciatus*, *Ae. aegypti* and *An. stephensi* were found after 24 h exposure, respectively (Tables 4, 5). Interestingly, AC-CE did not cause any larval mortality at LC_50_ and LC_90_ concentrations found for AC-AgNPs.

**TABLE 4:** Larval toxicity of AC-AE against larval stages of *Cx. quinquefasciatus*, *Ae. aegypti* and *An. stephensi* after 24h, 48h and 72 h exposure.

Time	LC_50_	95% CI	LC_90_	95% CI	Regression equation^a^	χ^2^ (*p*-value)
*Culex quinquefasciatus*						
24 h	231.41	200.01–308.77	524.35	450.81–703.41	*y* = −1.16 + 0.004*x*	3.31 (0.85)
48 h	188.71	161.69–214.03	431.49	362.87–539.18	*y* = −0.92 + 0.005*x*	2.84 (0.78)
72 h	147.50	123.61–170.73	391.20	334.12–507.03	*y* = −0.76 + 0.005*x*	3.91 (0.69)
*Aedes aegypti*						
24 h	110.33	85.44–214.11	160.14	141.00–348.11	*y* = −3.25 + 0.036*x*	8.44 (0.01)
48 h	90.30	71.80–109.37	141.42	123.02–241.88	*y* = −2.41 + 0.020*x*	5.15 (0.97)
72 h	71.52	66.27–100.11	113.11	91.76–199.44	*y* = −2.30 + 0.044*x*	4.01 (0.17)
*Anopheles stephensi*						
24 h	53.15	47.33–60.01	121.88	87.14–199.01	*y* = −2.88 + 0.050*x*	5.86 (0.001)
48 h	41.57	37.51–50.43	102.11	75.55–111.77	*y* = −3.36 + 0.081*x*	8.30 (0.32)
72 h	37.23	28.83–52.68	57.01	41.15–63.52	*y* = −2.52 + 0.070*x*	0.71 (0.15)

*Control* no larval mortality recorded; *LC*_*50*_ *, LC*_*90*_ Lethal concentration of the substance that kills 50%, 90% of the exposed larvae, respectively; LC_50_ and LC_90_ are expressed in µg/mL; *95% CI*, Confidence interval at 95%; *χ* ^*2*^ Chi square; Statistical significance was set at *p*-value <0.05.

^a^ Determined using the probit model.

**TABLE 5:** Larval toxicity of AC-AgNPs against larval stages of *Cx. quinquefasciatus*, *Ae. aegypti* and *An. stephensi* after 24h, 48h and 72h exposure.

Time	LC_50_	95% CI	LC_90_	95% CI	Regression equation^a^	χ^2^ (*p*-value)
*Culex quinquefasciatus*						
24 h	18.41	11.75–21.02	24.35	19.11–38.96	*y* = −9.16 + 0.59*x*	62.31 (<0.0001)
48 h	8.97	6.27–10.60	17.22	11.44–19.52	*y* = −7.32 + 0.52*x*	57.14 (<0.0001)
72 h^b^	—	—	—	—	—	—
*Aedes aegypti*						
24 h	16.71	15.86–17.53	24.16	20.98–27.59	*y* = −2.27 + 1.36*x*	7.15 (0.52)
48 h	7.52	5.81–9.42	16.63	15.54–17.97	*y* = −1.35 + 1.50*x*	10.3 (0.24)
72 h^b^	—	—	—	—	—	—
*Anopheles stephensi*						
24 h	10.67	7.59–13.75	21.62	12.49–28.76	*y* = −3.58 + 1.48*x*	5.35 (0.48)
48 h	5.85	3.75–8.94	12.06	10.55–19.80	*y* = −5.35 + 2.35*x*	8.30 (0.32)
72 h^b^	—	—	—	—	—	—

*Control* no larval mortality recorded; *LC*_*50*_ *, LC*_*90*_ Lethal concentration of the substance that kills 50%, 90% of the exposed larvae, respectively; LC_50_ and LC_90_ are expressed in µg/mL; *95% CI*, Confidence interval at 95%; *χ* ^*2*^ Chi square; Statistical significance was set at *p*-value <0.05.

^a^ Determined using the probit model.

^b^ No data were computed as all larvae were dead after 48 h

### 3.9 Behavioral and morphological impact of the AC-AgNPs on the larvae

**FIGURE 8:**
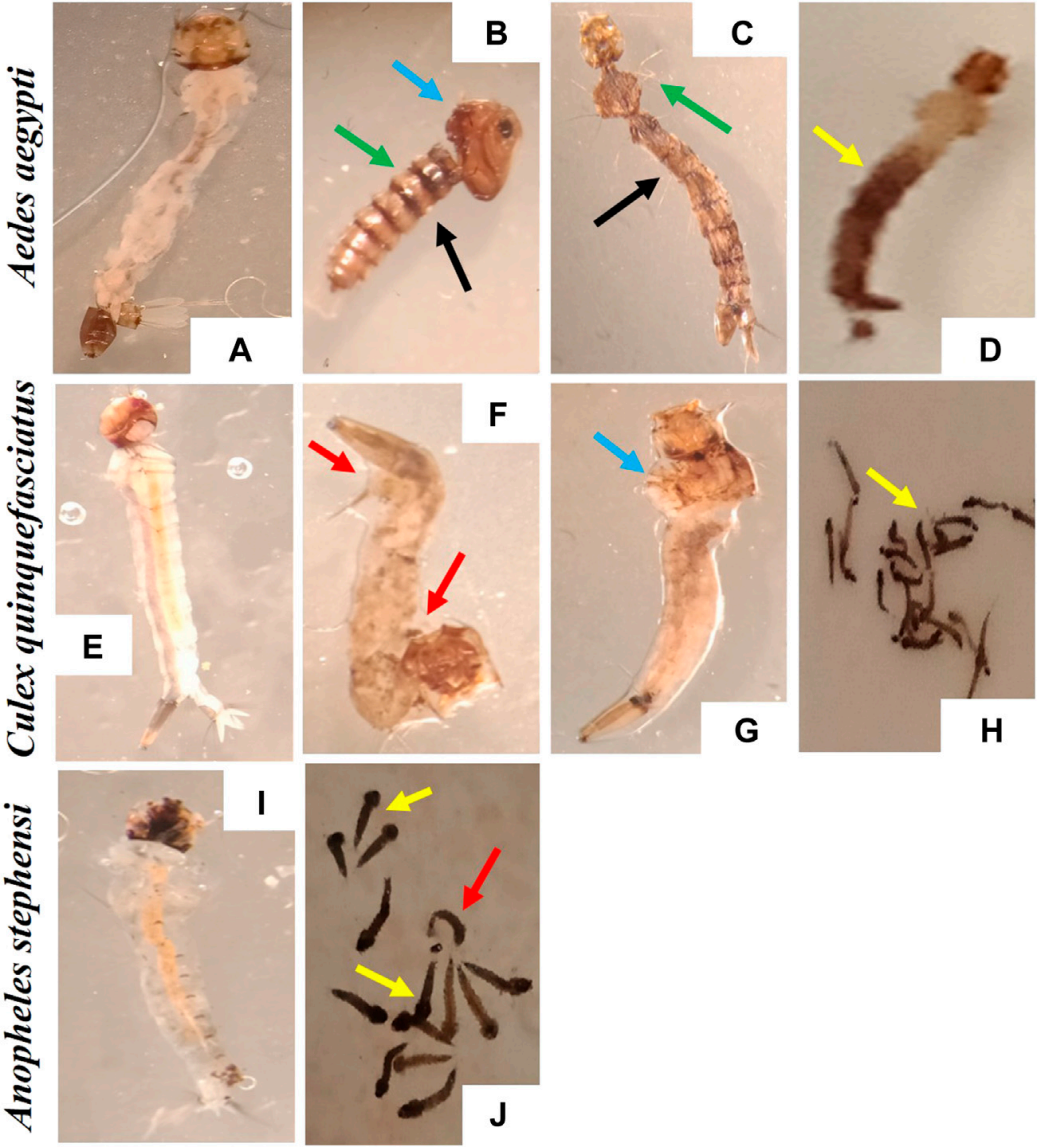
Morphological deformities induced by the exposure to AC-AgNPs (LC_50_ dose) on larval stages of *Ae. aegypti*, *Cx. quinquefasciatus*, and *An. stephensi*. **(A)** *Ae. aegypti* larvae (Control), **(B–D)** *A. aegypti* larvae (AC-AgNPs-treated), **(E)** *Cx. quinquefasciatus* larvae (Control), **(F–H)** *Cx. quinquefasciatus* larvae (AC-AgNPs-treated), **(I)** *An. stephensi* larvae (Control), **(J)** *An. stephensi* larvae (AC-AgNPs-treated). Arrows indicate the difference morphological abnormalities seen in AC-AgNPs-treated larvae: swelling of the apical cells (blue arrows), pigmentation of body (yellow arrows), shrinkage of the larvae (red arrows), loss of external anal and head hairs/bristles (green arrows), necrosis and thickening of the epidermis (black arrows).

The stereomicroscopic observations of *Ae. aegypti*, *An. stephensi* and *Cx. quinquefasciatus* larval stages treated with AC-AgNPs are depicted in Figure 8, and revealed the induction of behavioral and morphological changes in mosquito larvae. It was observed that swimming behavior of larvae was reduced, with morbid larvae at the bottom of bowls and unable to swim to the surface. Several morphological changes were noted in AC-AgNPs-treated larvae and these included loss of external hairs/bristles, swelling of the apical cells, pigmentation of the body, shrinkage of the larvae, and necrosis and thickening of the epidermis (Figure 8).

## 4 Discussion

Vector-borne diseases such as malaria are an important public health problem throughout the world especially in Cameroon. This study demonstrated good hemocompatibility and high biocidal potential of green synthesized AgNPs using *A. cordifolia* leaves (Euphorbiaceae).

The synthesis of AC-AgNPs through green route was rapid as color change was noted a few minutes after mixing AC-CE and AgNO_3_ aqueous solutions, thereby outlining the onset of AC-AgNPs synthesis through reduction of Ag^+^ ions into Ag⁰. This observation was further confirmed upon analysis of UV-Vis spectra with a peak at 445 nm wavelength. Karthik and others showed a close value (434 nm) for *Acalypha indica*, another Euphorbiaceae plant (Karthik et al., 2017). The UV-Vis peak corresponds to SPR phenomenon during which electron on NPs surface enter into resonance with the wavelength of incident light (Kojom Foko et al., 2021). The SPR band was increasing with parameters used for optimizing AC-AgNPs synthesis (i.e., AgNO_3_ concentration, AC-CE volume, incubation time and incubation temperature), and such findings were seen previously with plants growing in Cameroon, especially *Megaphrynium macrostachyum* (Eya’ane Meva et al., 2016), and *Selaginella myosurus* (Belle Ebanda Kedi et al., 2018).

The biofabricated AC-AgNPs were small and mostly spherical which is consistent with earlier reports using *Morinda citrifolia* and *Adiantum raddianum* (Suman et al., 2015; Govindarajan et al., 2017a). Using a systematic review, we previously reported that the bulk of NPs tested against *Plasmodium* parasites and mosquito vectors were spherical with a large range of size (Kojom Foko et al., 2019). Also, the nucleation theory of NPs synthesis suggests that slow rate of seed formation is expected to lead to broad size distribution of NPs (Liu et al., 2020). This result suggests that AC-AgNPs nucleation process was heterogeneous, and this can be influenced by several factors such as mixing time and solvation dynamics (Thanh et al., 2014; Deshpande et al., 2021). Size and shape of green NPs are modulated by complex interactions of plant- and experiment condition-related factors, and are crucial parameters that determine their physico-chemical and biological activities (Pal et al., 2007; Adams et al., 2014). Based on TEM analysis, AC-AgNPs were polydispersed with varied size. Such variation is commonly seen in AgNPs fabricated with plant extracts (Kojom Foko et al., 2019; Kojom Foko et al., 2021).

The analysis of PXRD and SAED patterns outlined that AC-AgNPs were polycrystalline with a crystallinity percentage of 84.13% and presence of additional peaks on diffractogram. This finding outlines that biosynthesized AC-AgNPs were not totally pure. At nanoscale level, a large number of metals present as face-centered cubic structures and tend to agglomerate due to high tension surface of ultrafine NPs (Belle Ebanda Kedi et al., 2018), thereby explaining the crystalline nature of AC-AgNPs. Also, with increasing nucleation and growing over time, NPs form twinned structures that then multiply with their surfaces bounded to cubic facets with the lowest binding energy (Annamalai and Nallamuthu, 2016). The SAED pattern clearly confirmed the crystalline nature of AC-AgNPs.

Silver atom was mainly involved in AC-AgNPs synthesis while other atoms such as oxygen and chlorine were also found, and these could be due to phytochemical compounds in AC-CE. FTIR spectrum revealed the presence of several peaks corresponding to functional chemical groups (e.g., O-H, C≡N, C=C) attributable to alkanoids, terpenoids, flavonoids, phenols, steroids, anthraquonones or saponins, and confirmed results from GC-MS-based phytochemical analysis done here and reported elsewhere (Osadebe et al., 2012). These compounds are likely involved in reducing silver ions during NPs synthesis along with their capping and stabilization (Hawadak et al., 2022). We found two predominant compounds in plant extract (2-hexadecen-1-ol, 3,7,11,15-tetramethyl-, [R-[R*,R*-(E)]]- (acyclic diterpene alcohol) and phytyl tetradecanoate (fatty acid phytyl ester) using GC-MS. Although mechanism of action of NPs reduction is uncertain, it is likely these compounds, alone or in combination with other compounds in plant extract, were involved in reduction, capping and stabilization of AC-AgNPs.

Zeta potential defines the stability of colloidal suspensions such NPs, and is a common parameter used to surface charge on a particle. In this study, zeta potential of AC-AgNPs was −18.1 mV. This value indicates a good stability of AC-AgNPs in dispersion medium. Indeed, negative surface charge is due to the binding affinity of AC-CE compounds with the NPs, conferring stability of AC-AgNPs and preventing several phenomena such as aggregation, sedimentation or flocculation which are known impair stability of particles (Faisal et al., 2021).

High lethal activity of green AC-AgNPs against *Pf* strains 3D7 and RKL9 was observed, with IC_50_ < 10 μg/mL for 3D7 and IC_50_ < 20 μg/mL for RKL9. This is consistent with value reported by Hawadak *et al.* and Rajkumar *et al.* using green NPs mediated by *Eclipta prostrata* and *Azadirachta indica*, respectively (Rajakumar et al., 2015; Hawadak et al., 2022). In contrast, our values are lower than those found previously with different *Plasmodium* strains (Kojom Foko et al., 2019). This antiplasmodial activity exhibited by the AC-AgNPs is due to above mentioned phytochemical compounds which served as bioreactors for NPs reduction and capping. Several studies suggested potential mechanisms of action of NPs against *Plasmodium* parasites (Cox-georgian et al., 2019; Abubakar et al., 2020). The NPs could induce parasite death by acting on several targets including cell membrane, enzymes and internal organelles (Shakeel et al., 2016; Kamaraj et al., 2017; Varela-Aramburu et al., 2020). Using *in vivo* model, Karthik and others showed that antiplasmodial activity of marine actinobacterial-mediated gold NPs was associated with increased production of tumor growth factor but reduction in tumor necrosis factor, thereby emphasizing an immunomodulatory role of NPs (Karthik et al., 2013). *Pf* is highly prevalent in Cameroon (Kojom Foko et al., 2018; Antonio-Nkondjio et al., 2019; Kojom Foko et al., 2021), and our findings suggest that AgNPs could be interesting as antimalarial drug. A large number of NPs-related chemical and/or physical factors could explain discrepancies obtained between our findings and those from previous studies. These included mainly size distribution, shape, capping/reducing agents, aggregation and surface charge. Even though AC-AgNPs synthesized in this study showed broad size distribution (range 6–28 nm), these are still interesting for future antimalarial drug development. Optimal NPs size for integration into human drugs varies depending on the specific drug and its intended application (Mitchell et al., 2021). This size distribution found here is consistent with previous studies on potential of MNPs as either drug delivery agent (i.e., passive targeting to enhance the accumulation of drugs in tumors) or antimalarial drug (i.e., active targeting to specific cells/tissues) (Santos-Magalhães and Mosqueira, 2010; Rahman et al., 2019). It should be interesting to conduct more studies to define consistent NPs size cut-offs for antimalarial therapy purposes.

It is known that antimalarial drugs such as ACTs, the current medicines used for treating uncomplicated malaria in most of endemic countries, can induce hemolysis in patients (Rehman et al., 2014). Therefore, new antimalarial drug candidates should be screened for hemocompatibility profile. The hemolysis rate was below at 50% after 30 minute-incubation, thereby underlining a HC_50_ > 500 μg/mL for the AC-AgNPs. The biofabricated AC-AgNPs were therefore highly hemocompatible, consistent with findings of Hossain and coworkers, who reported HC_50_ of 700 and 800 μg/mL for green aqueous and ethanolic NPs mediated by *Andrographis paniculata* stem (Hossain et al., 2019). Hemolysis increased as a function of time for AC-CE and AC-AgNPs which is in line with previous studies (Laloy et al., 2014; Avitabile et al., 2020). Hemolysis activity of NPs is strongly dependent on their size with higher hemolytic activity seen in smaller NPs (Chen et al., 2015). Thus, the small size of AC-AgNPs could likely explain their hemolytic activity (De La Harpe et al., 2019). Also, the anti-hemolytic activity of AC-AgNPs can be partially attributed to biomolecules coated on their surface. In fact, polyphenols are known to delay solubilization and inhibit oxidation of lipid frame; terpenes and flavonoids prevent interactions with hydrophobic parts of proteins and lipids, resulting in protecting and stabilizing cells membrane (Hoshyar et al., 2016; De La Harpe et al., 2019).

The phytofabricated AC-AgNPs exhibited a high toxicity against larval stages of *Ae. aegypti*, *Cx. quinquefasciatus* and *An. stephensi*, with LC_50_ below 20 μg/mL. Consisting with previous reports on diverse families of plants such as *A. raddianum* (Pteridaceae), *Hugonia mystax* (Linaceae), *Psidium guajava* (Myrtaceae), *Holostemma ada-kodien* (Apocynaceae) and *Aganosma cymosa* (Apocynaceae) (Govindarajan et al., 2017a, 2017b; Benelli and Govindarajan, 2017; Alyahya et al., 2018; Ntoumba et al., 2020). In contrast, some authors reported LC_50_ > 20 μg/mL for AgNPs fabricated with *Ventilago maderaspatana* (Rhamnaceae), *Naregamia alata* (Meliaceae), *Hedychium coronarium* (Zingiberaceae) and *Sargassum wightii* (Sargassaceae) (Azarudeen et al., 2017a, 2017b; Kalimuthu et al., 2017; Murugan et al., 2017). The discrepancy observed between studies is likely due to a cocktail of factors including the phytochemical composition of plant used for NPs synthesis, size/shape of NPs and mosquito strains. The mechanisms through which NPs induce larval mortality are still elusive, but it is thought that nanosized materials such as NPs can easily pass through insect exoskeleton and cell membrane, bind to sulphur-containing proteins and/or DNA which then lead to interference with homeostatic and physiological processes essential for larvae (e.g., copper homeostasis, osmoregulatory and spiracle-related respiratory systems) (Armstrong et al., 2013; Kojom Foko et al., 2021; Araújo et al., 2022). Other authors reported NP-induced physical and molecular degradation of insect gut as additional death cause (Kalimuthu et al., 2017, 2016; Banumathi et al., 2017; Ishwarya et al., 2017; Suganya et al., 2019). Also, these putative mechanisms could also explain behavioral and morphological modifications in AC-AgNPs-treated larvae seen in this study and by several earlier studies on extracts and NPs (Banumathi et al., 2017; Ishwarya et al., 2017; Suganya et al., 2019).

## 5 Conclusion

In this study, we synthesized, optimized, characterized and evaluated some medical applications of green AC-AgNPs including antiplasmodial, hemocompatibility and larvicidal potential. The synthesis was rapid and the optimized AC-AgNPs were mostly spheroidal, small-sized, dispersed, stable and polycrystalline in nature. Several phytochemicals including alkanoids, terpenoids, flavonoids, phenols and steroids were responsible for reduction, capping and stabilization of AC-AgNPs. The AC-AgNPs exhibited higher antiplasmodial and mosquito larvicidal activities compared to plant extract. The AC-AgNPs induced several mortality-associated behavioral and morphological changes in larval stages of *Ae. aegypti*, *An. stephensi* and *Cx. quinquefasciatus*. Finally, the AC-AgNPs exhibited good hemocompatibility with HC_50_ > 500 μg/mL. In worrying context of resistance of malaria parasites to current drugs and mosquitoes to different classes of insecticides, green nanotechnology could be a valuable and cutting-edge alternative for advanced drug/insecticide development and research.

## Data Availability

The original contributions presented in the study are included in the article/Supplementary Material, further inquiries can be directed to the corresponding author/s.

## Glossary

AC: Alchornea cordifolia
ACT: Artemisinin based combination therapy
AgNPs: Silver nanoparticles
ANOVA: Analysis of variance
a.u: Arbitrary units
CE: Crude extract
CI: Confidence interval
Cos: Cosinus
CQ: Chloroquine
DDT: Dichlorodiphenyltrichloroethane
DLS: Dynamic light scattering
DNA: Deoxyribonucleic acid
EDX: Energy dispersive X-ray
ELISA: Enzyme-linked immunosorbent assay
FCC: Face centered cubic
FTIR: Fourier transformed infrared spectroscopy
FWHM: Full width at half maximum
GC-MS: Gas chromatography coupled with mass spectrometry
HC50: 50% hemolysis concentration
IC50: 50% inhibition concentration
ICMR: Indian Council of Medical Research
JCPDS: Joint Committee on Powder Diffraction Standards
LC: Lethal concentration
MNPs: Metallic nanoparticles
NIMR: National Institute of Malaria Research
PBS: Phosphate-buffered saline
Pf: Plasmodium falciparum
PI: Polydispersity index
PXRD: Powder X-ray diffraction
RBC: Red blood cell
SAED: Selected area electron diffraction
SD: Standard deviation
SEM: Scanning electron microscopy
SPR: Surface plasmon resonance
TEM: Transmission electron microscopy
UD: The University of Douala
UV-Vis: Ultraviolet-Visible
WHO: World Health Organization
