# Supplementary material for: Phytofabrication and characterization of Alchornea cordifolia silver nanoparticles and evaluation of antiplasmodial, hemocompatibility and larvicidal potential
Source: Front Bioeng Biotechnol. 2023 Feb 28;11:1109841. doi: 10.3389/fbioe.2023.1109841 (PMC10011455; doi:10.3389/fbioe.2023.1109841)
Supplement: Supplementary file 1 [file DataSheet1.docx]

Supplementary Material


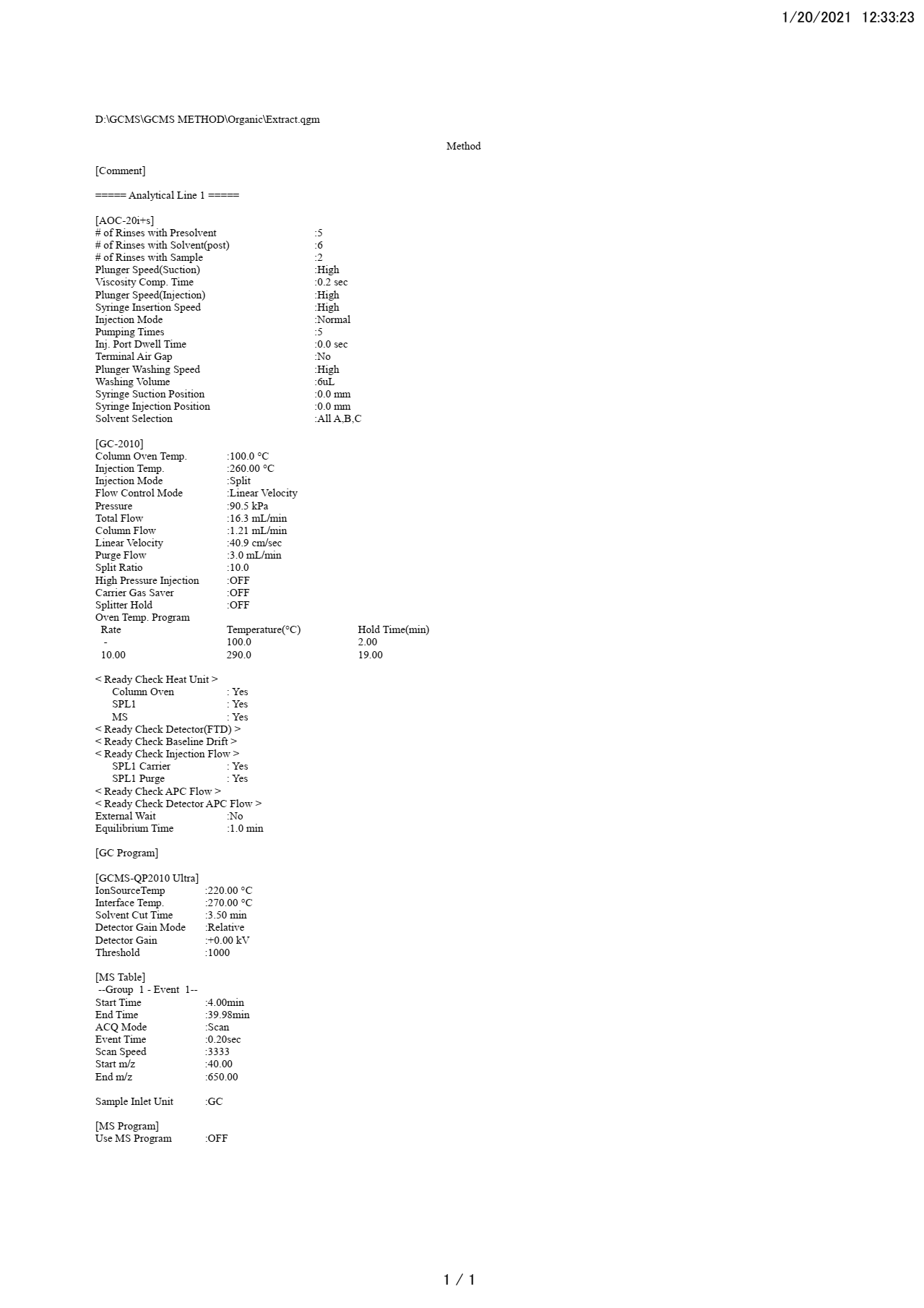


**Supplementary Figure 1**. Operational details of GC-MS analysis


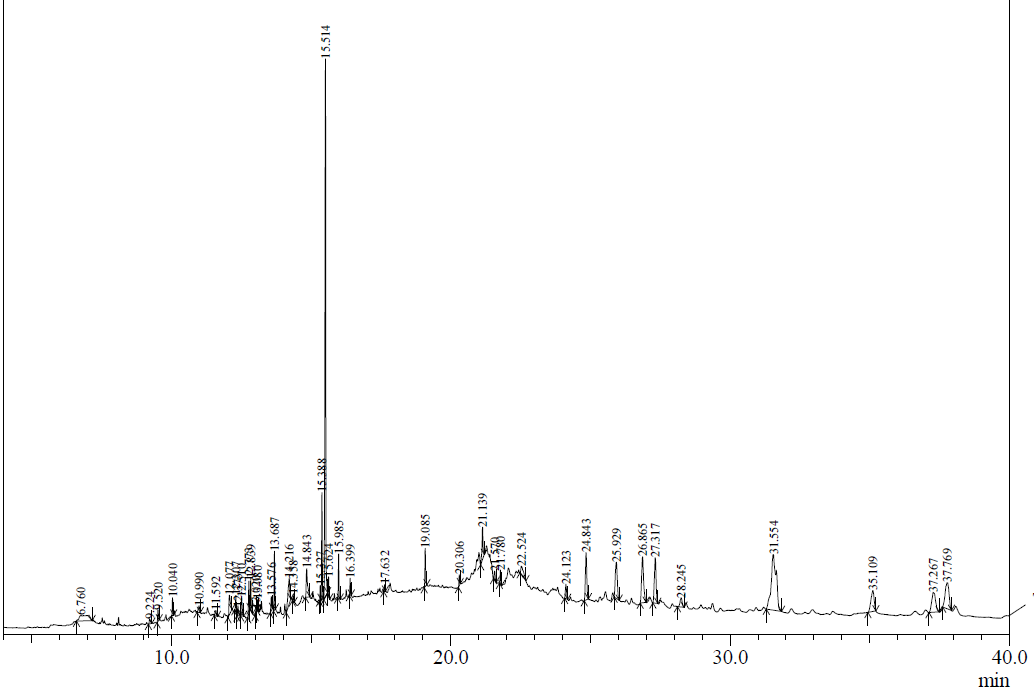


**Supplementary Figure 2**. GC-MS chromatogram of AC-CE


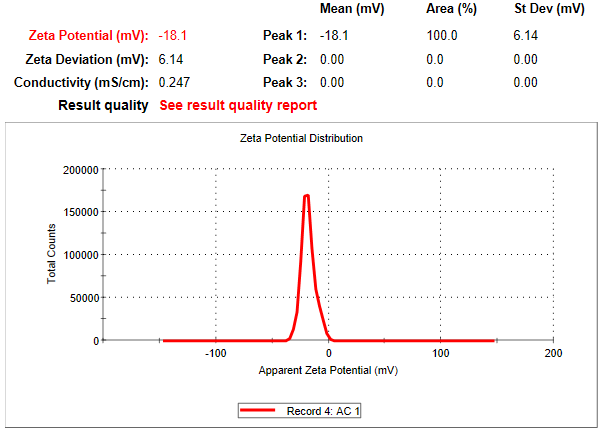


**Supplementary Figure 3**. Zeta potential of the *Alchornea cordifolia* silver nanoparticles


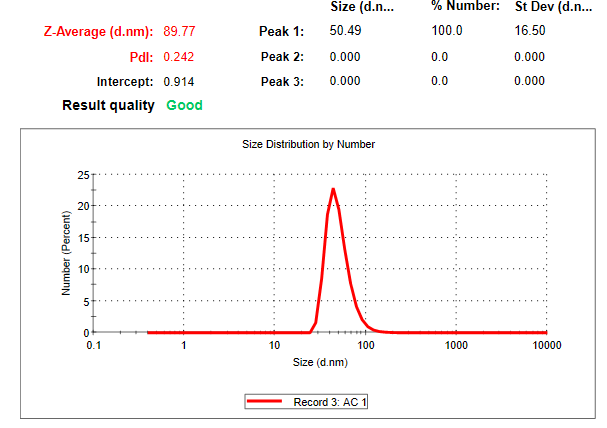


**Supplementary Figure 4**. Dynamic light scattering analysis of *Alchornea cordifolia* silver nanoparticles


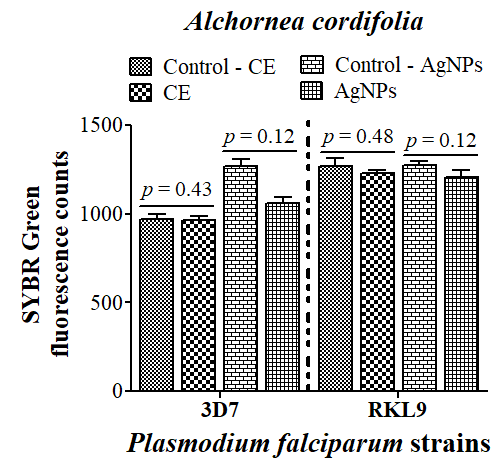


**Supplementary Figure 5**. Quenching effects during of the fluorescence-based antiplasmodial assay

**Supplementary Figure 6**. Mortality induced by AC-AgNPs against larval stages of (A) *Culex quinquefasciatus*, (B) *Aedes aegypti*, and (C) *Anopheles stephensi*. AC-AgNPs: *Alchornea cordifolia* silver nanoparticles, McNemar’s chi square test was used to compare mortality percentage. For a same concentration, the comparisons were made between 24h, 48h and 72h, and bars with the same letter are not statistically significant at *p* < 0.05
